# Impacts of climate-related hazards on health and well-being of vulnerable groups in Europe

**DOI:** 10.25646/10395

**Published:** 2022-08-31

**Authors:** Aleksandra Kazmierczak

**Affiliations:** European Environment Agency, Copenhagen, Denmark

Human-induced climate change is causing widespread impacts on human health and well-being. However, the magnitude of these impacts varies among individuals, communities and locations, and is driven by a combination of vulnerability and exposure. For example, older people, children, people experiencing poverty, and those in bad health or living with disability tend to be more vulnerable to climate change impacts than the general population. In addition, people’s ability to avoid, or cope with, climate hazards depends on their financial resources, extent of their social networks, home ownership, and other factors. Exposure is the likelihood of coming into contact with climate hazards, e.g., through residence in flood risk area, living in an easily overheated house, or through one’s occupation. Individuals and communities can often simultaneously experience different aspects of vulnerability and be exposed to various climate-related hazards; these compounding factors increase the chances of negative impacts on their health and well-being [1].

The unprecedented rise in temperatures since the 1990s, combined with the ongoing urbanisation, population ageing and disease prevalence in Europe, results in high and increasing exposure of vulnerable populations to heat. In particular in south and south-eastern Europe, the regions with lower socioeconomic status populations or higher percentages of elderly people correspond to areas affected by high temperatures. In European cities, nearly half of healthcare and educational facilities are in locations with a strong Urban Heat Island effect. In addition, approximately ten percent of schools and eleven percent of hospitals across Europe are in potential flood-prone areas [2].

The climate adaptation measures in place do not benefit all people to the same extent. For example, the most vulnerable groups tend to have lower access to green space and are least able to pay for flood insurance or flood-proofing of their homes. Thus, without consideration of equity in adaptation, the existing inequalities may be reinforced, or new inequalities may arise [2].

Although EU and national climate policies draw attention to vulnerable groups [3] and emphasise the need for equitable adaptation solutions, the practical implementation of such solutions remains scarce. Examples include development of heat health action plans with the most vulnerable groups in focus; targeted urban greening actions; or built environment improvements [2].

Ensuring that no one is left behind requires a meaningful engagement of vulnerable groups and the social care and health sectors in adaptation. At the same time, increased awareness of climate change among public health professionals and medical practitioners is crucial. Further, involvement in addressing climate change impacts of public administration, civil society and private sector from various fields, including public health, but also spatial planning or construction is needed to enable development of equitable adaptive actions [2].
